# Genomic sequence analysis of *Dissulfurirhabdus thermomarina* SH388 and proposed reassignment to *Dissulfurirhabdaceae* fam. nov.

**DOI:** 10.1099/mgen.0.000390

**Published:** 2020-06-17

**Authors:** Lewis M. Ward, Emma Bertran, David T. Johnston

**Affiliations:** ^1^​ Department of Earth and Planetary Sciences, Harvard University, Cambridge, MA, USA; ^2^​ Earth-Life Science Institute, Tokyo Institute of Technology, Tokyo, Japan; ^3^​ Department of Geosciences, Princeton University, Princeton, NJ, USA

**Keywords:** *Deltaproteobacteria*, sulfate reduction, comparative genomics, EET

## Abstract

Here, we report the draft genome sequence of *Dissulfurirhabdus thermomarina* SH388. Improved phylogenetic and taxonomic analysis of this organism using genome-level analyses supports assignment of this organism to a novel family within the phylum *Desulfobacterota*. Additionally, comparative genomic and phylogenetic analyses contextualize the convergent evolution of sulfur disproportionation and potential extracellular electron transfer in this organism relative to other members of the *Desulfobacterota*.

## Data Summary

The datasets generated and analysed during the current study are available in the National Center for Biotechnology Information repository under BioProject number PRJNA579145. Raw sequencing data is available in the SRA database under accession number SRR11035951, while the assembled genome is available in the WGS database under accession number JAAGRR000000000.

**Impact Statement**

Our understanding of Earth history is largely based on analysis of the rock record, but the preservation of chemical signals in rocks is modulated by the interaction of sediments with microbial metabolisms, many of which are poorly understood. The ways in which microbes are able to breathe insoluble compounds such as elemental sulfur is of particular interest. *Dissulfurirhabdus thermomarina* is a bacterium in the phylum *Desulfobacterota*, and is notable for its ability to perform extracellular electron transfer to respire insoluble compounds, as well as its ability to disproportionate elemental sulfur – essentially making a living by fermenting sulfur into sulfide and sulfate in a manner analogous to brewer’s yeast fermenting sugar into alcohol and carbon dioxide. Here, we report the genome of *Dissulfurirhabdus thermomarina* and show that it evolved its metabolic traits independently from other groups of bacteria capable of similar processes. This has relevance for understanding the biochemical mechanisms, diversity and evolution of both extracellular electron transfer and sulfur disproportionation, enigmatic metabolisms that are important for shaping the preservation of chemical signals in the rock record.

## Introduction

The disproportionation of intermediate valence sulfur species (converting substrates such as sulfite, thiosulfate and elemental sulfur into products of sulfate and sulfide) is a poorly understood microbial metabolism that has been implicated in the production and preservation of extremely depleted sulfur isotope signatures in the rock record [1, 2]. Despite its potential significance for understanding paleo-redox proxies [3], this metabolism is not well elucidated [4] and the genetic capacity for sulfur disproportionation is largely indistinguishable from that for sulfate reduction based on sequence data alone [5]. Mechanisms for elemental sulfur disproportionation are poorly understood, yet this process is of significant interest, particularly due to its ability to utilize an insoluble extracellular substrate. Most of the diversity of organisms shown to be capable of disproportionating elemental sulfur are members of the family *Desulfobulbaceae* of *Desulfobacterota* (formerly *Deltaproteobacteria *) [4]. However, a small number of distantly related organisms are also capable of elemental sulfur disproportionation, although it remains unknown whether these lineages use the same or different pathways for sulfur disproportionation, and whether these pathways have evolved convergently or whether their distribution is the result of a single evolutionary innovation followed by horizontal gene transfer. In order to resolve these possibilities, we report here the draft genome sequence of *Dissulfurirhabdus thermomarina* SH388. This organism was first isolated from a shallow marine hydrothermal vent and was shown to be capable of disproportionating sulfur compounds (including elemental sulfur and sulfite) [6], but is of uncertain phylogenetic affinity within the *Desulfobacterota* and is not closely related to elemental sulfur disproportionators within the *Desulfobulbaceae *. Understanding the genetics and evolutionary history of sulfur metabolism in *Dissulfurirhabdus thermomarina *, in contrast to members of the *Desulfobulbaceae *, may therefore provide insight into the diversity, mechanism and evolution of sulfur disproportionation metabolisms more broadly.

## Methods

Genome sequencing and analysis followed methods described previously [7, 8], and summarized here. Purified genomic DNA of strain DSM100025 was ordered from the DSMZ (Deutsche Sammlung von Mikroorganismen und Zellkulturen) and submitted to MicrobesNG for sequencing. Cultures were grown anaerobically at 50 °C on medium 1210a (* Thermosulfurimonas* medium) with strain-specific modifications, before genomic DNA extraction with a JetFlex genomic DNA purification kit (Genomed). DNA libraries were prepared with a Nextera XT library prep kit on a Hamilton Microlab Star automated liquid handling system. Libraries were sequenced on an Illumina HiSeq using a 250 bp paired-end protocol. Reads were adapter trimmed using Trimmomatic 0.30 [9] and *de novo* assembly was performed using SPAdes version 3.7 [10]. Annotation was performed using rast v2.0 [11]. Genome completeness was estimated with CheckM v1.0.12 [12], and likelihood of presence or absence of metabolic pathways was estimated with MetaPOAP v1.0 [13]. The taxonomic assignment of the genome was verified with GTDB-Tk v0.3.2 [14]. Hydrogenase proteins were classified with HydDB [15].

Phylogenetic analyses followed methods described previously [16, 17], and summarized here. Additional *Desulfobulbales* genomes were downloaded from the National Center for Biotechnology Information (NCBI) GenBank and WGS databases. Protein sequences used in analyses (see below) were identified locally with the tblastn function of blast+ [18], aligned with muscle [19] and manually curated in Jalview [20]. Positive blast hits were considered to be full length (e.g. >90 % the shortest reference sequence from an isolate genome) with *E* values greater than 1×10^−20^. Phylogenetic trees were calculated using RAxML [21] on the CIPRES science gateway [22]. Transfer bootstrap support values were calculated by booster [23], and trees were visualized with the Interactive Tree of Life viewer [24]. Taxonomic assignment was confirmed with GTDB-Tk [14]. Histories of vertical versus horizontal inheritance of metabolic genes were inferred by comparison of organismal and metabolic protein phylogenies [25–27].

## Results

The *Dissulfurirhabdus thermomarina* genome was sequenced at ~85× coverage as 508 907 reads. The assembled genome was recovered as 413 contigs (292 >500 nt, 194 >2500 nt) for a total of 2 536 728 nt with 70.82 mol% G+C and an N50 of 14 884. It encodes 2791 coding sequences and 53 RNAs. The genome was estimated by CheckM to be 96.75 % complete.

## Discussion

**Fig. 1.**
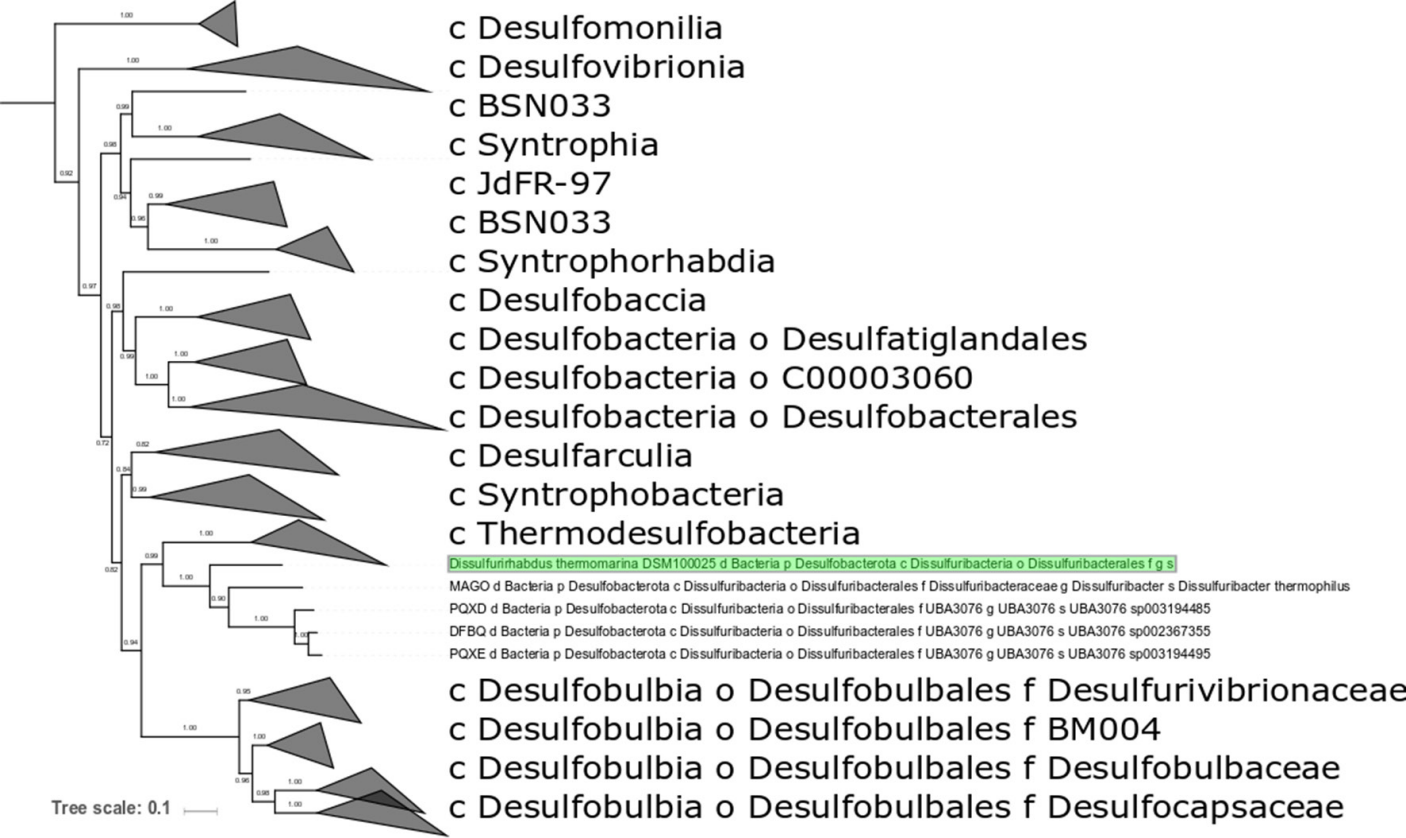
Phylogeny of the *Desulfobacterota*, showing the placement of *Dissulfurirhabdus thermomarina* relative to other lineages, built with concatenated ribosomal proteins following the methods of Hug *et al*. [38]. Strains are labelled with NCBI WGS database accession numbers and/or taxonomic assignments made with GTDB-Tk [14]. Scale bar represents average number of substitutions per site.

When first isolated, *Dissulfurirhabdus thermomarina* was placed in the *Deltaproteobacteria *, but was not assigned at lower taxonomic ranks. Following genome-wide taxonomic analysis with GTDB-Tk, *Dissulfurirhabdus thermomarina* is robustly placed in the order *Dissulfuribacterales* (phylum *Desulfobacterota*, class *Dissulfuribacteria*), but does not cluster with any characterized families within this order (Fig. 1). *Dissulfurirhabdus thermomarina* is sufficiently divergent from its closest characterized relative*, Dissulfuribacter thermophilus*, to suggest that these organisms represent separate families within the *Dissulfuribacterales*. Therefore, we propose assignment of *Dissulfurirhabdus thermomarina* as the type species of a novel family, *Dissulfurirhabdaceae*, within the order *Dissulfuribacterales* of *Desulfobacterota*.

**Fig. 2.**
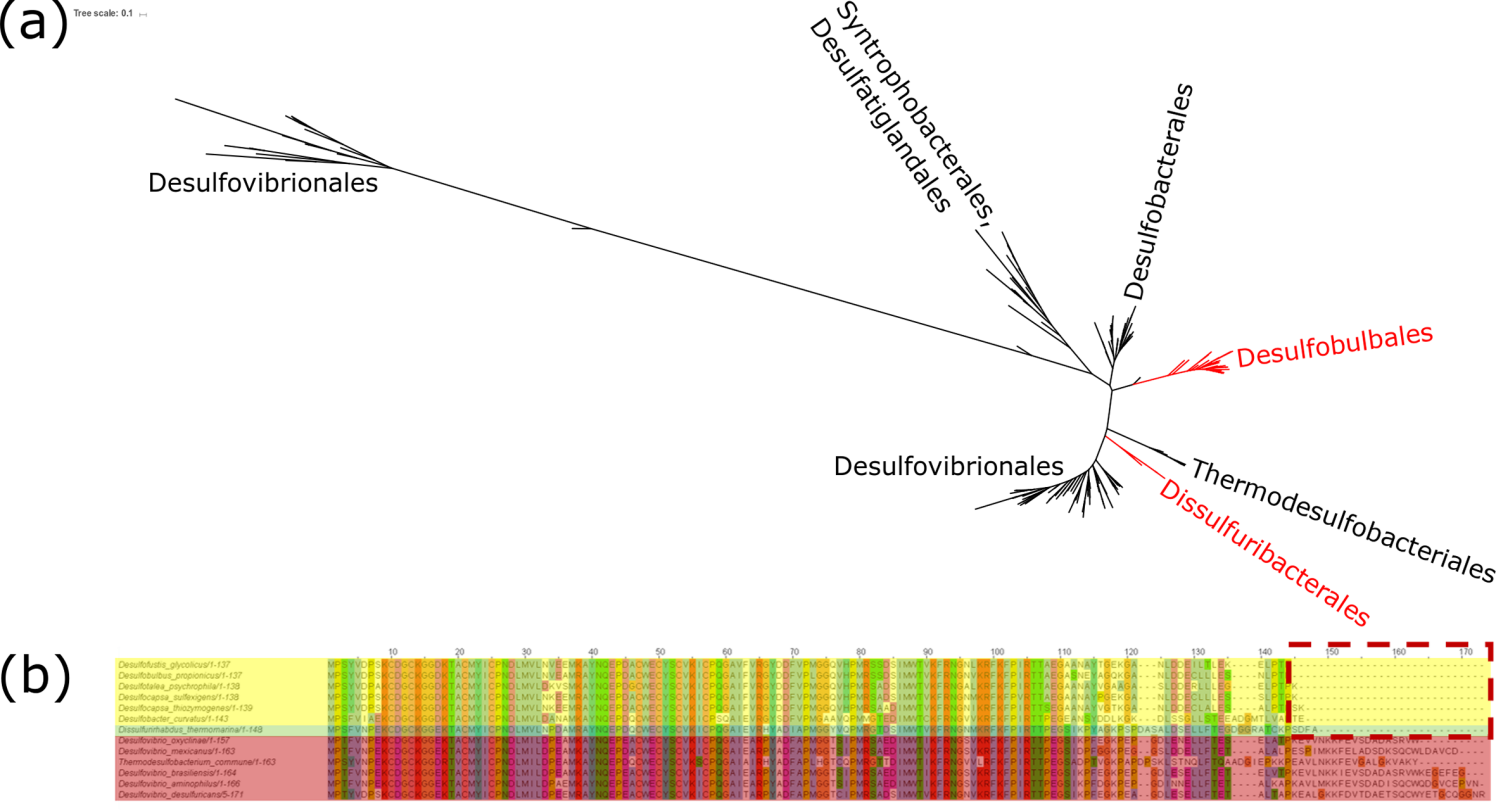
(a) Phylogeny of AprB proteins from members of the *Desulfobacterota*, with sequences from the *Dissulfuribacterales* (including *Dissulfurirhabdus thermomarina *) and members of the *Desulfobulbales* highlighted in red. These groups include sulfur disproportionators that encode AprB proteins with a truncated tail, but they are not closely related and are separated by many lineages of non-disproportionating bacteria that encode full-length AprB proteins; therefore, it appears that these traits have convergently evolved in the two lineages. Scale bar represents average number of substitutions per site. (b) Multiple sequence alignment of AprB proteins showing the truncated tail (dashed red box) present in members of the *Desulfobulbales* (highlighted in yellow) and *Dissulfurirhabdus thermomarina* (highlighted in green), but not in other lineages such as *Desulfovibrionales* or *Thermodesulfobacteriales* (highlighted in red).

Sulfur disproportionators are expected to encode the same marker genes as sulfate reducers ([5]; consistent with this expectation, *Dissulfurirhabdus thermomarina* encodes sulfate adenylyltransferase, adenylylsulfate reductase, dissimilatory sulfite reductase and the complex DsrMKJOP, which is associated with sulfite reduction. Known sulfur disproportionators in the family *Desulfobulbaceae* of *Desulfobacterota* encode AprB proteins with a truncated tail; this conserved truncation has been proposed as a molecular marker of the capacity for sulfur disproportionation [28]. *Dissulfurirhabdus thermomarina* encodes an AprB protein with a similar truncation despite encoding an AprB protein that is only distantly related to those of the *Desulfobulbaceae* (Fig. 2) – this suggests that the AprB truncation evolved independently in *Dissulfurirhabdus thermomarina* and the *Desulfobulbaceae *. This reinforces hypotheses for the association between this marker and the capacity for disproportionation as previously proposed [28]. Moreover, the apparently independent acquisition of the AprB truncation and the capacity for disproportionation in *Dissulfurirhabdus thermomarina* and members of the *Desulfobulbaceae* suggests that this might be a more widespread trait that has evolved convergently multiple times in ancestrally sulfate-reducing lineages. The relatively small number of physiologically and genomically characterized sulfur disproportionators available at this time limits our ability to confidently test this association, however, and so will necessitate further consideration as more data becomes available.

*Dissulfurirhabdus thermomarina* is capable of autotrophic growth [6] and encodes CO dehydrogenase/acetyl-CoA synthase, suggesting it makes use of the reductive acetyl-CoA (Wood–Ljungdahl) pathway like many sulfate-reducing bacteria [29]. The genome encodes a hydrogenase annotated by HydDB as a group 1 c NiFe hydrogenase associated with anaerobic respiratory uptake of H_2_. *Dissulfurirhabdus thermomarina* does not encode canonical proteins for aerobic respiration or denitrification, consistent with its inability to use O_2_ or nitrate as electron acceptors in culture [6]. However, this organism encodes a cytochrome *bd* O_2_ reductase; while these enzymes can in some cases be coupled to aerobic respiration (e.g. in *Nitrospira)* [30], they are often found in obligate anaerobes [31] in which they are likely used for O_2_ detoxification and oxidative stress tolerance [32].

*Dissulfurirhabdus thermomarina* is capable of disproportionating not only soluble sulfur species such as sulfite, but also insoluble elemental sulfur [6]. The mechanism of elemental sulfur disproportionation in *Dissulfurirhabdus thermomarina* and other strains capable of this metabolism is not known yet, but likely involves novel extracellular electron transfer pathways. Extracellular multihaem cytochrome proteins are commonly involved in respiratory electron transfer to insoluble mineral substrates [33, 34], and could, therefore, be expected to be involved in disproportionation of elemental sulfur. Analysis of the *Dissulfurirhabdus thermomarina* genome with CXXCH_finder [33] recovered 81 proteins with haem-binding domains, including one hypothetical protein with 26 haem-binding motifs – on par with proteins from bacteria such as *Geobacter* and *Shewanella *, well known for their capacity for extracellular electron transfer and ability to respire extracellular mineral substrates [33]. In addition to haem-binding motifs, this hypothetical protein also encodes seven cytochrome *c*_3_ motifs associated with extracellular iron respiration in *Shewanella* [35]. This hypothetical protein shows low similarity to other proteins accessible on the NCBI database (<50 % to any sequence), but of all proteins from well-characterized organisms it is most similar to a hypothetical protein from *Thermosulfidibacter takaii *, a thermophilic bacterium capable of elemental sulfur reduction. The putative extracellular electron transfer protein from *Dissulfurirhabdus thermomarina* also has notable similarity (~30 %) to extracellular iron oxide respiratory system periplasmic decahaem cytochrome *c* protein components from *Shewanella oneidensis* MR-1, including the protein DsmE associated with respiration of extracellular substrates [36]. Therefore, we propose that *Dissulfurirhabdus thermomarina* utilizes extracellular multihaem cytochrome proteins related to those in dissimilatory iron-reducing bacteria in order to transfer electrons to insoluble substrates such as elemental sulfur. In contrast, members of the *Desulfobulbales* that are characterized as being capable of elemental sulfur disproportionation (e.g. *Desulfobulbus propionicus *, *Desulfocapsa thiozymogenes *) encode proteins with no more than 11 or 12 CxxCH motifs. However, it remains possible that the capacity for extracellular electron transport has convergently evolved in diverse lineages of sulfur disproportionators, but has so far remained undiagnosed. It has recently been shown that some cable bacteria – members of the *Desulfobulbales* notable for their capacity for long distance and extracellular electron transfer – may also be capable of sulfur disproportionation [37]. It is therefore likely that the co-occurrence of sulfur disproportionation and extracellular electron transport metabolisms may have evolved independently in multiple lineages of sulfate-reducing bacteria, potentially utilizing diverse mechanisms.

### Conclusions

In addition to better constraining the phylogenetic placement and taxonomy of this organism, the draft genome of *Dissulfurirhabdus thermomarina* provides evidence for the evolutionary history of metabolic pathways it employs. This includes the apparent convergent evolution for the capacity for sulfur disproportionation in different lineages of *Desulfobacterota* (e.g. *Dissulfurirhabdus* and members of *Desulfobulbaceae *). These organisms appear to have evolved sulfur disproportionation via similar genetic mechanisms independently from an ancestral state of sulfate reduction. The presence of putative multihaem cytochrome proteins encoded by *Dissulfurirhabdus thermomarina* also suggests that the capacity for elemental sulfur disproportionation may in some cases utilize mechanisms homologous to those used in the respiration of extracellular electron acceptors such as metal oxides, as has been well studied in organisms such as *Geobacter* and *Shewanella *, demonstrating the versatility of micro-organisms to adapt proteins encoded in their genomes and acquired via horizontal gene transfer in order to develop novel metabolic traits.

## Data Bibliography

Ward, LM, E Bertran, DT Johnston; NCBI BioProject; PRJNA579145 (2020).

Ward, LM, E Bertran, DT Johnston; NCBI WGS; JAAGRR000000000 (2020).

Ward, LM, E Bertran, DT Johnston; NCBI SRA; SRR11035951 (2020).
